# Very Late Local Relapse of Ewing's Sarcoma of the Head and Neck treated with Aggressive Multimodal Therapy

**DOI:** 10.1155/2008/854141

**Published:** 2008-05-29

**Authors:** J. Thariat, A. Italiano, F. Peyrade, I. Birtwisle-Peyrottes, L. Gastaud, O. Dassonville, A. Thyss

**Affiliations:** ^1^Department of Radiation Oncology, Centre Antoine Lacassagne, 33 avenue de Valombrose, 06189 NICE Cedex 2, France; ^2^Department of Cytogenetics, Centre Antoine Lacassagne, 33 avenue de Valombrose, 06189 NICE Cedex 2, France; ^3^Department of Medical Oncology, Centre Antoine Lacassagne, 33 avenue de Valombrose, 06189 NICE Cedex 2, France; ^4^Departement of Pathology, Centre Antoine Lacassagne, 33 avenue de Valombrose, 06189 NICE Cedex 2, France; ^5^Department of Head and Neck Surgery, Centre Antoine Lacassagne, 33 avenue de Valombrose, 06189 NICE Cedex 2, France

## Abstract

Ewing's sarcoma's relapse rarely occurs more than two years after the initial diagnosis. We report the case of a 26-year-old man with a history of Ewing's sarcoma of the left maxillary sinus at the age of 10 who presented with a very late local relapse, 16 years after the first occurrence of disease. Ultimate control was achieved after multimodal therapy including surgery, high-dose chemotherapy, and radiotherapy. This report indicates that local relapses of Ewing's sarcoma can be treated with curative intent in selected cases.

## 1. INTRODUCTION

Ewing's sarcoma usually occurs before 30 with a peak incidence at 15 years of age. It represents 5% of all primary bone tumors and is the second most common malignant bone tumor in children. It is extremely rare in the head and neck as it represents 3% of osseous Ewing's sarcoma (OES) [1]. The mandible is most frequently affected. Only six cases of maxillary sinus OES have been reported to date [2]. Children are usually omitted radiation therapy as long as complete resection can be achieved to avoid radiation-induced growth defects and the risk of radiation-induced sarcomas [3]. Late relapses have seldom been described [4–6]. We report here an observation corresponding, to the best of our knowledge, to the longest period of complete remission followed by local relapse thus far described and discussed its clinical implications.

**Case 1.**

A 26-year-old man self-referred for isolated moderately paced painless swelling of the left nasal ala with no physical status deterioration. His past medical history was remarkable for Ewing's sarcoma of the left maxillary sinus at the age of 10 (stage T2N0M0 and IIA with radical margins according to the American Joint Committee on Cancer (AJCC) and Musculoskeletal Tumor Society (MSTS) staging systems, resp.). The patient had been treated according to the Société Française d’Oncologie Pédiatrique (SFOP's) protocol with chemotherapy, hemimaxillectomy and palatine prosthetic reconstruction, and postoperative chemotherapy without radiation therapy because of the absence of residual viable tumor cells in the tumor resection specimen. The protocol was adapted from the SFOP guidelines and consisted of (1) initial chemotherapy with cyclophosphamide-doxorubicin, (2) radical surgery if possible (children were stratified for chemotherapy and radiation on histological response: patients with less than 10% tumor cells on resection specimen and clear margins (complete resection) were considered good responders and were omitted local radiotherapy), and (3) maintenance chemotherapy (for up to 9 months) with actinomycin-vincristine.

**Figure 1:**
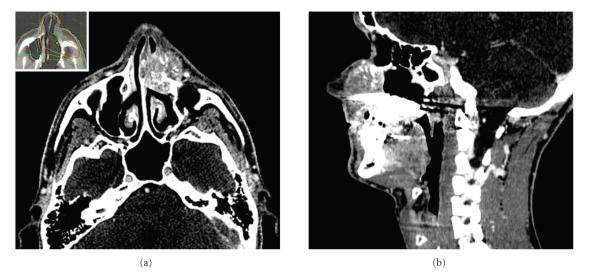
CT scan showing a contrast-enhanced lesion with central sunburst pattern and calcifications black asterisk (upper (axial view), lower (sagittal view)) was noted in the para-nasal and cheek soft-tissues. It extended medially into the surrounding nasal bone structures and posteriorly into the left maxillary sinus and nasal fossa. It extended superiorly to palatine prosthesis black square. Insert on Figure 1(a) shows conformational radiotherapy plan (according to Euro-Ewing 99 recommendations). Initial tumor volume was treated with a 2 cm margin and included posttherapeutic scar tissue area white asterisk.

**Figure 2:**
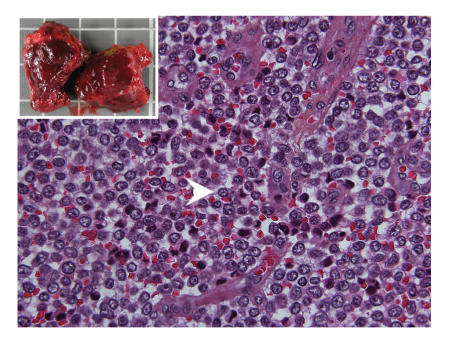
(Histological examination) HES × 600 small-round-blue-cell arrow head tumor with highly cellular areas. Insert (gross examination) showed a pseudoencapsulated fleshy tumor mass.

Swelling had worsened over the past three months and discharge appeared over the past month. Examination was consistent with a large mucocele of the left nose area. On computerized tomography (CT) scan, a 19-mL contrast-enhanced lesion with central sunburst pattern and calcifications (Figure 1) was noted in the para-nasal and cheek soft-tissues. It extended medially into the surrounding nasal bone structures and posteriorly into the left maxillary sinus and nasal fossa. Caldwell Luc operation revealed a fleshy purple lesion. The lesion was easily resectable from subcutaneous soft tissues but was largely extending into the frontal process of the surrounding maxillary bone. Resection was therefore suboptimal (macroscopically incomplete). Grossly, the lesion appeared as a purple matter with areas of hemorrhage and necrosis (Figure 2). Histologically, it was a small-round-blue-cell tumor with homogenous highly cellular areas spread apart by conjonctival septa and hemorrhagic lakes. Cells had round hyperchromatic nuclei and scant cytoplasm. Histology was consistent with the diagnostic of Ewing's sarcoma. A molecular cytogenetic analysis was performed. R-banded metaphase cells showed a characteristic t(11;22)(q24;q12) translocation. A rearrangement of the EWSR1 (22q12) gene was detected by fluorescence in situ hybridization with a specific locus probe. These findings confirmed the histological diagnosis of Ewing's sarcoma. Whole body workup including thoracic and abdominal CT scan, multiple bone marrow biopsies, and aspirates showed no additional abnormality (stage rT1N0M0 at relapse). Chemotherapy consisted of four cycles of doxorubicin ifosfamide followed by intensification with high-dose busulfan-cyclophosphamide with autologous peripheral-blood stem cell rescue. Complete response to chemotherapy was achieved. Conformational radiation therapy (54 Gy) was prescribed according to Euro Ewing 99 protocol [7] due to both suboptimal resection at relapse and recurrent disease in the adulthood. The patient is alive and well with no evidence of disease 15 months after diagnosis of relapse.

## 2. DISCUSSION

Multimodality therapy for Ewing's sarcoma is associated with markedly improved survival rates. Surgery followed by adjuvant radiotherapy and multiagent chemotherapy has dramatically improved five years survival rates to 70%, compared with single—or even dual—modality therapy [8]. In our case, the patient had not been treated with radiotherapy at initial diagnosis because of the absence of residual disease following surgical resection. The choice of local control treatment modalities depends on several factors including tumor site, tumor size, and age [9]. Survival rates in patients with Ewing's sarcoma of the head and neck may be better than those of patients with tumors in other locations but vary depending on the bone involved [10] and the possibility to perform surgery. Complete surgical excision is undertaken whenever possible as nonsurgical treatment is associated with a poorer long-term survival rate than that of a treatment regimen that includes surgery. Radiotherapy has been used historically for the treatment of Ewing's sarcomas, as those sarcomas are relatively radiosensitive [11]. However, a significant proportion of children treated with radiotherapy develop late toxicity, including growth defects and occurrence of second malignancies [3]. Therefore, radiotherapy is not recommended for children in whom surgical resection can be performed with clear margins. However, most treatment failures in patients who do not have distant metastases at presentation result from local recurrence. Moreover, adjuvant radiotherapy has been associated with improved local control and improves survival when compared to surgery or radiotherapy alone [9, 12]. Finally, it might be questioned whether radiotherapy should have been advocated initially in our patient. As there was no evidence for any microscopic disease after dose-intense chemotherapy and complete surgery, it was estimated that the benefit of increased local control in this case would have exceeded the risk of toxicity.

Early metastatic relapse has the poorest prognosis [13]. Collins' law states [14] that for mesenchymal tumors of the childhood, the period of risk for tumor recurrence is the age of the child at diagnosis plus nine months. On the other hand, 85% of the relapses occur within three years [13, 15]. However, some relapses occur later than five years after initial diagnosis. Of those, metastatic relapses are by far the most common [4–6, 16]. One case only of regional relapse has been described 17 years after diagnosis in one series [5]. To our knowledge, no such late strictly local relapse in the head and neck area as in our case has been reported so far. Such rare occurrences may be misdiagnosed. Had our patient not been particularly aware of his past medical history and given the mucocele-like aspect of his relapse in the head and neck area, he might have not been treated adequately in nonspecialized centers. Such late local relapse might question whether the patient had in fact a late relapse of his initial tumor or a clonally distinct recurrence. The most reliable method to answer this question would have been to analyze the fusion gene sequence in both the primary and relapsed tumor pathologic specimens. Unfortunately, initial pathologic specimens were not available in our case.

Five-year survival after relapse is less than 10% but selected patients benefit from aggressive salvage treatment [13]. In particular, late relapses may be treated with curative intent as in our case. Indeed, patients with longer time to first recurrence represent the subset of patients most likely to survive following recurrence [15] provided that the diagnosis is not delayed to a point, where disease is no longer manageable with optimal maximum dose-intensity treatment.

Finally, the patient is well and free of disease 15 months after relapse. While followup following treatment of relapse is still short, intensive salvage multimodality treatment including radiotherapy for local relapse was feasible and ultimate tumor control was achieved.

## 3. CONCLUSION

Such past medical history as Ewing's sarcoma must not be overlooked when facing apparently benign tumor since very late local relapse is possible. Ultimate control can be achieved for local relapses of Ewing's sarcoma in the adulthood with aggressive salvage treatment protocols. Further data are needed to assess the role of aggressive local treatment.
